# A systems approach to clinical oncology: Focus on breast cancer

**DOI:** 10.1186/1477-5956-4-5

**Published:** 2006-04-04

**Authors:** Mark Abramovitz, Brian Leyland-Jones

**Affiliations:** 1 Center for Clinical Research in Oncology and Department of Oncology, McGill University, 546 Pine Avenue West, Montreal, Quebec, H2W 1S6, Canada

## Abstract

During the past decade, genomic microarrays have been applied with some success to the molecular profiling of breast tumours, which has resulted in a much more detailed classification scheme as well as in the identification of potential gene signature sets. These gene sets have been applied to both the prognosis and prediction of outcome to treatment and have performed better than the current clinical criteria. One of the main limitations of microarray analysis, however, is that frozen tumour samples are required for the assay. This imposes severe limitations on access to samples and precludes large scale validation studies from being conducted. Quantitative reverse transcriptase polymerase chain reaction (qRT-PCR), on the other hand, can be used with degraded RNAs derived from formalin-fixed paraffin-embedded (FFPE) tumour samples, the most important and abundant source of clinical material available. More recently, the novel DASL (cDNA-mediated Annealing, Selection, extension and Ligation) assay has been developed as a high throughput gene expression profiling system specifically designed for use with FFPE tumour tissue samples.

However, we do not believe that genomics is adequate as a sole prognostic and predictive platform in breast cancer. The key proteins driving oncogenesis, for example, can undergo post-translational modifications; moreover, if we are ever to move individualization of therapy into the practical world of blood-based assays, serum proteomics becomes critical. Proteomic platforms, including tissue micro-arrays (TMA) and protein chip arrays, in conjunction with surface-enhanced laser desorption ionization time-of-flight mass spectrometry (SELDI-TOF/MS), have been the technologies most widely applied to the characterization of tumours and serum from breast cancer patients, with still limited but encouraging results.

This review will focus on these genomic and proteomic platforms, with an emphasis placed on the utilization of FFPE tumour tissue samples and serum, as they have been applied to the study of breast cancer for the discovery of gene signatures and biomarkers for the early diagnosis, prognosis and prediction of treatment outcome. The ultimate goal is to be able to apply a systems biology approach to the information gleaned from the combination of these techniques in order to select the best treatment strategy, monitor its effectiveness and make changes as rapidly as possible where needed to achieve the optimal therapeutic results for the patient.

## Background

In the United States it is estimated that approximately 213,000 new cases of invasive breast cancer will be diagnosed in 2006 and 41,000 women are expected to die from this disease [1]. Breast cancer will account for ~31% of new cancer cases among women in the United states in 2006 [1]. Current treatment strategies rely mainly on anatomic staging that continues to play a significant role in the decision making process. Classical pathological indexes that are used to predict survival, development of metastatic disease or guide selection of primary therapy in patients with breast cancer include the Nottingham Prognostic Index [2], introduced back in 1982, Adjuvant! Online (AO) [3], and the St. Gallen criteria [4]. The Nottingham Prognostic Index is based upon tumour size, lymph node stage and histological grade to predict survival in patients. AO is a program that is available through the web that is used to assess risk for the development of metastatic disease using traditional prognostic factors that include age, lymph node (LN) status, tumour size, tumour grade, and hormone receptor status. The current St. Gallen derived algorithm for selection of adjuvant systemic therapy for early breast cancer patients includes tumour size and grade, nodal status, menopausal status, peritumoural vessel invasion, endocrine status and HER2 (epidermal growth factor receptor 2) status. The use of these aforementioned mainly pathology-based prognostic tools in the treatment decision-making process are inadequate and, at minimum, a more precise stratification of patients into responders versus non-responders to therapeutic agents is needed.

Although large clinical trials have confirmed the value of systemic therapy, it is not possible to identify at the outset those patients who are likely to respond to adjuvant treatment or which types of treatment should be used. For example, adjuvant therapy significantly improves survival in breast cancer patients with both LN-negative and LN-positive disease. It is generally accepted that breast cancer patients with a poor prognosis would gain the most benefit from adjuvant therapy (e.g. those with invasion into axillary LNs). However, results of several studies show that 22 to 33% of breast cancer patients with no detectable LN involvement and classified into a good prognosis subgroup develop recurrence of disease after a 10-year follow-up. Therefore, accurate identification and classification of breast cancer patients over and above current prognostic and predictive markers is critically important for rational treatment decision-making and improved clinical outcome in the individual patient, a long time goal, albeit an elusive one, of breast cancer researchers.

Multiple chemotherapeutic agents that include anthracyclines, antimetabolites, microtubule inhibitors, and alkylating agents have been used successfully in the treatment of breast cancer. Although, response rates of >30% are routinely achieved in previously untreated patients it is still not possible to know which of these cytotoxic therapies will be the most effective treatment for a particular patient's tumour. Once treatment begins, a patient is monitored for response and toxicity and any change in therapy is solely based upon tumour progression or intolerable toxicity. Even in the case of targeted therapies in breast cancer that hit the estrogen receptor (ER), HER2 or EGFR (human epidermal growth factor receptor 1), the ability to effectively monitor response is critical. Thus, despite the development of numerous general and targeted anti-cancer agents over the past two decades, more progress is needed with regard to increasing life expectancy once a woman is diagnosed with breast cancer [5]. Thus, the ability to diagnose breast cancer early, determine each woman's prognosis, predict the best treatment modality and effectively and non-invasively monitor the patient's progress to treatment is paramount if we are to move forward in our fight against breast cancer.

The way forward will require a systematic approach that will entail both genomics and proteomics if we are to meet the goal of therapeutic individualization. The reasons for this are several: 1) Molecular profiling by microarray, qRT-PCR or FISH- (fluorescent-*in situ *hybridization) based analyses of tumours is required for classification of tumour types that correlates with prognosis; 2) Analysis at the gene level, however, can not detect the biologic subtleties introduced through posttranslational modifications, glycosylations, etc. of proteins and thus requires a proteomic approach; 3) Analysis of serum samples for predictive markers can only be accomplished through proteomics; and 4) Older technologies (e.g., histopathology and immunohistochemistry) may still prove valuable for particular treatments in individual patients. For instance, a genomic lead suggesting amplification of a particular gene could be validated at the proteomic level or through immunohistochemistry. The path to individualization of therapy is complex and in the long run will require some combination of genomic and proteomic analysis in conjunction with existing histopathologic and immunohistochemical analysis in order to provide a complete picture of a patient's breast cancer. Only by analyzing data across technology platforms and using improving computational systems biology approaches will we be able to eventually define all of the pieces to the puzzle.

Microarray technology has allowed for unprecedented expression profiling of thousands of genes simultaneously and for the development of gene signatures to subdivide and categorize tumours [6] as well as predict response to treatment [7]. However, this platform relies on frozen tumour tissue samples which are not a readily available source; but other complementary platforms, such as TMA, qRT-PCR and the recently described DASL assay [8], will allow the researcher to tap into the large resource of well annotated FFPE tumour tissue sample repositories. Analysis of mRNA expression using any one of these above mentioned platforms, however, provides only a limited look at what is going on in a tumour since repeat biopsies are not a realistic option and, therefore, will not provide a means of following a patient's response to therapy. This will require the ability to analyze proteins in readily accessible serum samples from patients being treated with various drugs using recently developed protein-chip arrays in conjunction with SELDI-TOF/MS. The ability to monitor and predict a patient's response to therapy will allow any necessary changes to therapy be made as rapidly as possible. A systematic approach, therefore, will entail a precise pathological and molecular classification of the patient's tumour that would be used to select the best possible treatment strategy, monitor the effects of treatment in order to modify treatment as needed, and predict recurrence of disease.

This review will focus on this systematic approach and the application of existing and newer genomic and proteomic technologies, introduced above, as they apply to the study of breast cancer; approaches that will contribute to, and change the way in which we diagnose, treat, and monitor treatment effectiveness in patients.

## Classification of breast tumours using microarrays

Microarray-based gene expression profiling of human cancers has rapidly emerged as a new powerful screening technique. Recently, breast cancer gene expression signatures have been identified that are associated with ER and LN status of patients and can aid in classifying breast cancer patients into subgroups with different clinical outcomes. Moreover, gene expression signatures have been shown to predict response to particular chemotherapy regimens (see below). The advantage of the microarray technology is the ability to measure the RNA expression of thousands of genes at one time, and to relate how the expression pattern of one gene correlates to the expression of other genes in or between different tumour samples.

One of the first comprehensive attempts to characterize the variation in gene expression between sporadic breast tumour samples was published by Perou and co-workers [9]. This groundbreaking study was the first to establish that tumours could be phenotypically classified into subtypes distinguishable by differences in their expression profiles. An "intrinsic gene set" of 476 cDNAs was then used to cluster and segregate the tumours into four major subgroups: 1) a "luminal cell-like" group expressing ER; 2) a "basal cell-like" group expressing cytokeratins 5 and 17, integrin 4, and laminin, but lacking ER expression; 3) a HER2-positive subset; and 4) a "normal" epithelial group. Hence, this seminal paper identified specific subtypes of breast tumours based upon hormone receptor and HER2 status.

A subsequent study by the same group has extended the molecular profiling of breast cancer by applying their intrinsic gene set to cluster 78 cancers (the tumours from their previous study were included in these), 3 fibroadenomas, and 4 normal breast tissue samples [6]. In this study, they identified 456 genes (out of 8,102 candidate genes) that could classify breast tumours into six subtypes (including ER-positive, luminal subtype A; ER-positive, luminal subtype B; ER-positive, luminal subtype C; HER2-positive, ER-negative subtype; basal-like, ER-negative, progesterone receptor- (PGR) negative, HER2-negative; normal breast-like) and subsequently validated these subtypes in an independent cohort of 49 patients with locally advanced breast cancer. The authors found that these subtypes were highly significantly correlated with overall survival (OS) (or the percentage of subjects in a study who have survived for a defined period of time, usually from the time of diagnosis). A similar classification scheme was obtained for 99 LN-negative and LN-positive breast cancer patients [10]. ER status was the most important discriminator of subtypes with tumour grade being a distant second. In addition, subtypes did not strongly reflect other clinical features, such as LN status, tumour size or menopausal status, underscoring the importance of molecular characterization of tumours.

Van't Veer and colleagues [11] have used DNA microanalysis on primary breast tumours of 98 young patients and applied supervised classification to identify a 70-gene-expression signature strongly predictive of a short interval to distant metastasis in LN-negative patients <55 years of age. The poor prognostic signature consisted of genes regulating cell cycle, invasion, metastasis, and angiogenesis. Van de Vijver and co-workers [12] subsequently used this 70-gene prognostic profile to classify a series of 295 consecutive patients with primary breast carcinomas as having a gene-expression signature associated with either a poor or a good prognosis. All patients had stage I or II breast cancer and were younger than 53 years of age; 151 had LN-negative disease, and 144 had LN-positive disease. The predictive power of the prognostic profile was validated using univariate and multivariate statistical analyses. Among the 295 patients, 180 had a poor-prognosis signature and 115 had a good prognosis signature, and the overall 10-year survival rates were 55% and 95%, respectively. At 10 years, the probability of remaining free of distant metastases was 51% in the group with a poor-prognosis signature and 85% in the group with a good-prognosis signature. The estimated hazard ratio (or relative risk of an endpoint at any given time) for distant metastases in the group with a poor-prognostic signature, as compared with that for the group with the good-prognostic signature, was 5.1 (or 5.1 times greater). This ratio remained significant even when the groups were analyzed according to LN status. Multivariate Cox regression analysis showed that the prognosis profile was a strong independent factor in predicting disease outcome. The resulting gene-expression profile was a more powerful predictor of the outcome of disease in young patients with breast cancer than the 2001 St. Gallen criteria, or the NIH consensus criteria [13] based on clinical and histologic characteristics. In fact, use of these criteria resulted in a misclassification of a significant number of patients who would be either under-treated or over-treated with adjuvant therapy. Strikingly, their prognostic profile was independent of LN involvement but rather, was based upon its strong predictive power with respect to metastasis to non-lymphatic tissues.

Shortly thereafter, Piccart and colleagues [14] presented the validation of the Amsterdam 70-gene prognostic signature in LN-negative untreated breast cancer at the San Antonio Breast Cancer Symposium. This validation was performed as part of preparation for the launch of the large prospective randomized clinical trial, MINDACT, for LN-negative breast cancer, powered to look at the utility in clinical practice of the Amsterdam 70-gene prognostic signature. Significant heterogeneity between the Amsterdam and the external validation samples was found and is currently being investigated; however, the 70-gene prognostic signature outperformed both the Nottingham Prognostic Index and the St. Gallen criteria in predicting time to distant metastases and OS. In addition, women of the present series classified as low risk by the gene signature had a projected 5 year distant metastasis-free survival of 95%. While the overall performance of the 70-gene prognostic signature was inferior in this external validation series compared to the original Amsterdam series, the results provide evidence for the clinical value of this new genomic tool and are encouraging to mobilize forces for the conduct of MINDACT.

A number of recent microarray studies have been published which also deal with patient risk assessment in those with LN-negative breast cancer. In one such study, Wang and Co-Workers [15] analyzed primary tumour samples from 286 LN-negative patients of all age-groups and tumour sizes who had not received any adjuvant systemic treatment. They identified a 76-gene signature that showed 93% sensitivity and 48% specificity when tested in an independent set of 171 LN-negative patients. This set of genes was useful in identifying patients who developed distant metastasis within 5 years. By using this gene signature only 52% of low-risk patients would be recommended to receive systemic adjuvant chemotherapy compared with 90% by the St. Gallen criteria. However, the utility of this gene signature will have to be confirmed in a larger cohort of patients prior to being used to recommend that low-risk patients not be treated with unnecessary adjuvant systemic therapy. In another recent study, a 64-gene signature set was derived at by hierarchical clustering of microarray data from 159 tumour samples from both adjuvant-treated and untreated patients that identified genes associated with prognosis and impact of adjuvant therapies, defined as distant metastasis or death within 5 years [16]. This set of genes was then validated in an independent cohort of 289 patients and could be used to stratify patients into low, medium and high risk groups that outperformed current clinical criteria. Patients in the low risk group could potentially be spared adjuvant chemotherapy while those in the high risk group might benefit from aggressive therapies such as anthracyclines or taxanes, however, this would have to be validated in a larger prospective study.

## Use of microarray data to predict response to therapy

Other recent microarray studies have focused on determining gene signatures of potential response of patients to specific chemotherapy and hormonal therapy regimens. In one such study, Chang and co-workers [7] have shown that gene profiling can be used to accurately predict response to neoadjuvant docetaxel. The study enrolled 24 subjects from whom core needle breast tumour biopsies were taken. RNA was extracted from these biopsies and subjected to microarray analysis that resulted in the construction of a 92-gene predictor of response set. In a cross-validation analysis, the classifier correctly identified 10 of 11 responders and 11 of 13 non-responders for an overall accuracy of 88%. Correlation between RNA expression measured by the Affymetrix arrays and semi-quantitative RT-PCR was also ascertained. In addition, this classifier was validated in an independent set of 6 subsequent patients.

Similar results were achieved in a more recent study in which an 85-gene signature was selected from over 2,400 genes using a high-throughput RT-PCR technique [17]. Samples from 44 breast tumour tissues, taken prior to treatment, were analyzed and genes were selected based upon differential expression between responders and non-responders. They then devised a diagnostic system based upon a weighted algorithm and used it to predict the clinical response to docetaxel therapy in 26 patients with over 80% accuracy.

A second neoadjuvant study was recently published using cDNA arrays to develop a predictor of response to sequential paclitaxel and fluorouracil + doxorubicin + cyclophosphamide, involving 42 samples, 24 of which were used for predictive marker discovery and 18 of which were used as an independent validation set. A classifier with 74 markers was developed, with 78% accuracy, suggesting that transcriptional profiling has the potential to identify a gene expression pattern in breast cancer that may lead to clinically useful predictors of chemotherapy response [18]. A prospective clinical trial is currently underway to validate these initial findings. And finally, in a study published by Jansen and co-workers [19], a 44-gene signature was used to predict tamoxifen-responsive and tamoxifen-resistant tumours. They were able to predict tamoxifen-resistance with an accuracy of 77% compared with 50–60% accuracy based on ER status alone.

Additional work in ascertaining expression patterns for other commonly prescribed chemotherapy regimens, like anthracyclines and anti-metabolites, or targeted therapy, like trastuzumab, is underway in the hope that these patterns can be incorporated into predictive tests for the selection of an appropriate treatment to minimize toxicity and maximize efficacy for women with breast cancer. This technology, hence, offers the means of identifying potentially useful predictive clinical genes/gene signatures that, when validated, may reduce unnecessary chemotherapy treatment for women with breast cancer. In addition, these results compare very favorably with the best existing predictive factors for response to specific therapy, and strongly suggest that after appropriately extensive validation, these identified genes/gene signatures will be useful for treatment selection. One major caveat, though, is that the inability to routinely use FFPE tumour tissue samples in microarray analysis restricts the use of this technology to relatively small sample sets and requires validation of potential genes/biomarkers using additional genomic (qRT-PCR, DASL) and/or proteomic (TMA) platforms (dealt with in subsequent sections below).

## Use of FFPE tumour tissue for downstream analysis platforms

While the above mentioned microarray studies have relied upon freshly-frozen tissue samples, this material is difficult to collect for large scale studies, cumbersome to process and expensive to store long-term. In the past few years, enormous progress has been made in developing technologies to exploit FFPE tumour tissue samples for gene expression and proteomic analysis. FFPE tissue is the standard processing methodology practiced in pathology laboratories the world-over. These samples are highly stable at room temperature, are easily stored and, most importantly, make up a vast archive of pathologically well characterized and well annotated clinical samples from randomized trials that exist worldwide. FFPE tumour tissue samples are, therefore, an immense resource that are amenable for conducting both retrospective and prospective biomarker investigations which will allow for well-controlled hypothesis testing.

Several groups have illustrated that it is feasible to extract and purify RNA from such FFPE tissue and to perform gene expression profiling despite the chemical modification and often fragmentation of RNA that occurs due to the fixation process [[20-27]]. With the development of qRT-PCR technology, it is now possible to detect rare messages in FFPE tissue and to examine the variation of expression over quite a large dynamic range. Amplicons are designed specifically on small segments of DNA (less than 100 bps) to achieve close to 100% efficiency for all amplicons regardless of their length and nucleic acid composition. This is also ideal for fixed tissue since most RNA species in FFPE tissue are degraded to fragments that are ≥ 100 bp; as such, transcripts can be easily detected by oligonucleotide primers that span small amplicons of less than 100 bp. The potential of this technology, as pointed out above, becomes even more attractive in that it permits the analysis of thousands of well annotated archived tissue samples without the need to collect the relatively complicated freshly-frozen tissue. Platforms that can use this wealth of available samples should, therefore, shorten the time for biomarker development and validation and is currently being exploited in the Oncotype DX assay [28] (see below).

FFPE tumour tissue samples are routinely used for immunohistochemical analysis and with the development of TMA technology, large tissue sample sets can be used to evaluate the role of potential biomarkers as diagnostic or predictive tools in breast cancer (see below).

## Tissue Micro-Arrays for the evaluation of breast tumour biomarkers

**Figure 1:**
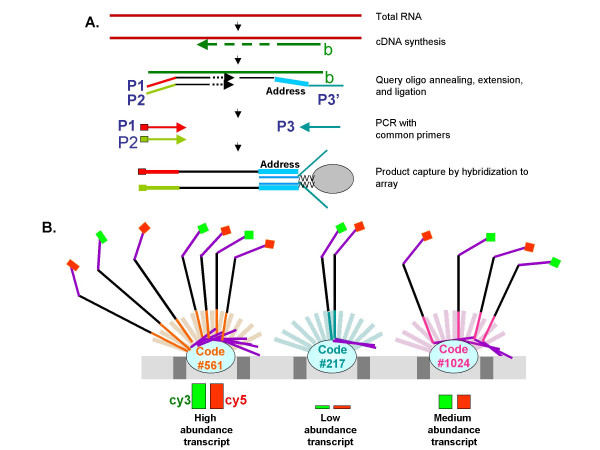
How the DASL Assay works. **A. **The DASL Assay monitors gene expression with probe groups to query total RNA-generated cDNA target sequences. Total RNA, prepared from FFPE tumour tissue, is converted to cDNA using both biotinylated (designated as b) random nonamers and oligo dT. Primer sites, incorporated within each probe group, are used for PCR amplification. Also within the probe group is an address sequence complementary to one of the 1536 sequences on the Sentrix^® ^Array Matrix. Three probe group per gene are used that allows for detection of small (1.3) fold-changes in expression between samples. Probe groups are annealed to the biotinylated cDNA, followed by selection of the duplexes on streptavidin beads to remove unhybridized oligos. Correctly annealed, assay specific oligos are extended and ligated to a locus-specific oligo. The locus-specific oligo incorporates an address sequence and primer site for the generation of amplifiable products. **B. **Address-containing templates, that are labeled with either cy3 or cy5 fluorescent dyes during PCR amplification, are subsequently hybridized to complementary address sequences immobilized to beads on the arrays. The array is then scanned to acquire intensity data.

In the TMA format, an array comprises hundreds of different patient samples using cores as small as 0.6 mm taken from FFPE tumour blocks [29]. Each array is incubated with one detection protein (i.e., antibody): a single analyte endpoint is measured and directly compared across multiple samples (Fig. 1). Probing multiple arrays with different specific antibodies provides the effect of generating a multiplex read-out. TMAs, thus, were developed as a means of identifying protein targets in as many as 1,000 cylindrical FFPE tissue specimens taken from individual tumours. This has opened the door to the analysis of protein expression using archival tissue, and TMAs provide a cost-effective method for examining multiple biomarkers on a large series of retrospective and prospective breast cancer cases. The TMA platform has been employed to explore a limited number of biomarkers that are potentially involved in malignant progression and tumour biology using similar analytical methods used to analyze microarray data.

The first application of this high-throughput technique was towards the identification of six genes amplified in breast cancer as well as p53 and ER in order to define novel subgroups [30]. However, in order for TMAs to gain broad acceptance, Camp and co-workers [31] showed that TMAs were basically immunohistochemically equivalent to whole tissue sections. They proved this by studying the expression of three common antigens in invasive breast carcinomas; ER, PGR and HER2. They found that analyzing two 0.6 mm cores from each tumour sample was comparable to the analysis of a whole tissue section in greater than 95% of cases. As importantly, they were able to perform TMAs on archival tissue dating back more than 60 years. Therefore, although tissue cores used in TMA studies are small compared with full sections, their effectiveness as tools in cancer studies has been rigorously validated over a short period of time by a number of investigators [[32-35]]. Since these initial publications on the development and use of TMAs in the analysis of tumours from breast cancer patients, this technique has been widely applied to the study of breast cancer both with regard to classification and tumour markers.

Callagy and colleagues [35] sought to validate microarray-derived classification schemes using FFPE tissue from well-annotated archival samples. A TMA study was undertaken to sub-classify 107 breast cancers using thirteen different putative protein biomarkers. Analysis, using an unsupervised clustering algorithm, subdivided the tumours into two main groups that correlated with tumour grade and nodal status. Not surprisingly, no single biomarker tested could identify these groups; classification, thus, requires the input of several proteins in order to subdivide tumours into clinically relevant subgroups. Based upon their 13 biomarkers, tumours could be subdivided into 2 main groups, ER-positive and ER-negative. ER-positive tumours fell into 2 subdivisions; subsets A and B both expressing luminal cytokeratins 8/18 but showing differential expression of HER2, c-Myc and PGR. Subset A was most reminiscent of luminal subtype A described using expression microarray analysis [6]. ER-negative tumours could be subdivided into subsets C and D, but only subset D expressed basal cytokeratins, suggesting that basal-type tumours form a distinct subset of ER-negative breast cancer. This data, therefore, supported the findings from previous microarray studies conducted on a limited number of samples [6,9]. However, this pilot study did not show any correlation between the tumour classification and survival, underscoring the need for determining the optimal set of biomarkers required to generate a robust classification system for breast cancer.

In two more recent TMA studies, described below, hierarchical clustering analysis did show prognostic significance. Makretsov and co-workers [36] evaluated 31 biomarkers in 438 invasive breast carcinoma cases with 15.4 years of follow-up. Of these 31 biomarkers, 17 were prognostically significant while an additional 4 showed a trend but did not reach significance. They then used these 21 immunomarkers to conduct unsupervised hierarchical cluster analysis and were able to segregate the samples into three prognostically significant cluster groups. This clustering could also be achieved with only 11 biomarkers (ER, PGR, HER2, p53, Ki67, CA IX, TIP1, stromal CD117, PTEN, p63 and CK5/6) which contributed significantly to their cluster group designation. Cluster group 1 was ER-positive and PGR-positive, while cluster groups 2 and 3 were, for the most part, negative for these steroid hormone receptors. With regard to survival, only cluster groups and LN status were significant as independent prognostic variables. Indeed, some tumours that expressed high levels of ER were classified as belonging to group 2 or 3, ER-negative groups. Thus, expression of biomarkers associated with tumour aggressiveness can override positive prognostic factors such as ER. It is especially important to be able to determine the nature of LN-negative breast cancer so that toxic chemotherapy is only administered to those patients that need it while sparing others that don't.

A larger TMA study was recently completed in which 1,076 invasive breast cancer cases were evaluated using 25 biomarkers [37]. Biomarkers used in the study were selected based upon; 1) their well established role that they play in breast carcinogenesis; 2) their ability to act as discriminator genes that can stratify breast cancer in to distinct groups, identified in microarray studies; and 3) their ability to identify specific forms of differentiation. Markers came from the following groups: luminal, basal, hormone receptors, EGFR family members, tumour suppressor genes, cell adhesion molecules, mucins, apocrine differentiation and neuroendocrine differentiation. Six main clusters were identified by hierarchical cluster analysis: Groups 1 and 2 were ER-positive, luminal, while groups 3 & 6 were HER2-positive, luminal. However, group 3 was distinguishable from group 6 based upon strong MUC1 and weak E-cadherin expression in group 3 and weak MUC1 and strong E-cadherin expression in group 6. Group 4 only contained 4 tumours which were all EGFR-positive but ER-negative. Finally, group 5 was basal, HER2-negative and ER-negative. This study clearly showed that apparent homogeneous tumours could be classified into biologically and clinically distinct groups and it confirmed the previous classification of breast cancer carried out by microarray analysis [6].

BRCA1 and BRCA2 tumours have also recently been subjected to TMA analysis in which 37 biomarkers were used to define hormone receptor, cell cycle, apoptosis and basal cell status [38]. The TMA contained cores from 20 BRCA1, 14 BRCA2 and 59 sporadic age-matched breast carcinomas. In agreement with previous work, BRCA1 showed phenotypic characteristics that were notably different from BRCA2. Analysis of all sporadic and hereditary cases by unsupervised hierarchical clustering revealed two main branches, an ER-positive and an ER-negative cluster. Within the second cluster, BRCA2 carcinomas were intermixed with sporadic tumours, however, most BRCA1 carcinomas, found in the first cluster, were grouped into a main sub-branch that included tumours that expressed basal cell markers and/or p53. There was a clear separation of this sub-group from HER2-positive sporadic carcinomas. In fact, by FISH analysis, HER2 was not amplified in any of the BRCA tumours but was amplified in 22% of the sporadic ones. In contrast to this, c-Myc was found to be amplified in 23% (3/13) of BRCA1 and 67% (4/6) BRCA2 tumours. With regard to individual markers, the most striking difference between BRCA1 and BRCA2 hereditary breast carcinomas was in expression of cell cycle proteins. BRCA2 tumours expressed cyclin D1 and D3, cyclin D kinase (CDK) 4 and the CDK inhibitors, p16, p21, and p27, which were all downregulated in BRCA1. BRCA1 could be characterized as having a basal phenotype, ER-negative and HER2-negative, with up-regulation of cyclin A and caspase 3, but downregulation of cyclin D1 and D3, CDKIs (p16, p21, p27), and BCL2, the opposite phenotype from most BRCA2 carcinomas.

TMAs have also been used to evaluate breast carcinomas with a limited number of biomarkers in order to test the hypothesis that a particular marker has biological and/or therapeutic significance in breast cancer. It has been known for some time now that the long term use of nonsteroidal anti-inflammatory drugs (NSAIDs) are linked to a 40–50% reduction in colon cancer [39]. The effect of NSAIDs on colon cancer is caused by inhibition of cyclooxygenase-2 (Cox-2) resulting in the blockade of synthesis of prostaglandins (PG), foremost PGE_2_, that promotes tumourigenesis, invasion and metastasis by stimulating angiogenesis and inhibiting immune surveillance [40]. However, the importance of Cox-2 in breast cancer has not been established. TMA analysis was performed on 200 breast carcinomas and Cox-2 was detected in 41% of cases [41]. Cox-2 expression was positively correlated with HER2 and Ki-67, a marker of proliferation, but was inversely correlated with expression of ER and PGR. A potential role of Cox-2 in breast cancer is based upon its role in proliferation [42], apoptosis [43] as well as angiogenesis [44]. Although a significant association of Cox-2 with disease-free survival (DFS) (or the survival period spanning the time from surgery to recurrence of cancer) was not achieved, Cox-2 still represents an interesting target in breast cancer [45].

Deacetylation of histones by histone deacetylases (HDACs) counteracts histone acetylation resulting in DNA that is inaccessible causing suppression of gene transcription. Over the past few years, inhibitors of HDACs have shown promise as potential anti-cancer agents [46], and have been found to inhibit cell growth and induce apoptosis in breast cancer cells [[47-50]]. A TMA study [51], using tumour samples from 200 breast cancer patients, was conducted that focused on HDAC-1 and HDAC-3, from HDAC class I, since these play a role in proliferation and cell survival of mammary tumour cells and can interact either directly or indirectly with the steroid hormone receptors ER and PGR as well as the tumour suppressor p53 [[52-54]]. They found that HDAC-1 was significantly associated with improved DFS but not with the classical prognostic factors. In a sub-group of ER/PGR-positive, HER2-negative tumours, expression of HDAC-1 was associated with a better DFS probability then HDAC-1 negative tumours. In general, HDAC-1 expression was linked to less aggressive tumours while those tumours in which HDAC-1 was not detected were more aggressive. Thus, HDAC-1 expression could be added to the list of potential markers of prolonged DFS and tumour aggressiveness.

Abnormal expression of a number of mucins, large glycoproteins expressed by many epithelial cells, has been implicated in many different cancers. Overexpression of MUC1 along with MUC2 and MUC3 has been detected in breast cancer [55]. The precise role that mucins play in cancer in general and breast cancer in particular has yet to be determined. In a study by Rakha and colleagues [56], expression of a number of mucins (MUC1, MUC2, MUC3, MUC4, MUC5AC and MUC6) were profiled by TMA in 1447 cases of invasive breast cancer in order to evaluate their prognostic significance. MUC1 and MUC3 were detected in the majority of breast cancer cases. Most strikingly, it was the subcellular localization of these two mucins and not their expression level that was of prognostic value; membranous staining being associated with poor OS compared with apical staining and a more favourable OS. The other mucins did not appear to have any prognostic value in predicting outcome.

One of the main objectives for the use of TMA technology is to identify prognostically relevant groups of breast cancer patients and, in conjunction with data from other molecular profiling studies, come up with an optimal panel of biomarkers that can be validated in independent sample sets. In the end, the aim of many of these studies is to be able to identify those biomarkers that can be used in a clinical setting to rapidly characterize a patient's tumour type and use that information to apply the most appropriate treatment strategy. Therefore, molecular classification of breast tumours will contribute important information to the traditional histopathologic classification currently in use. However, the use of TMAs requires a prior knowledge of the potential biomarkers being assessed and, therefore, can not readily contribute to the discovery of novel biomarkers.

## qRT-PCR: The Oncotype DX prognostic assay for tamoxifen-treated patients

QRT-PCR technology represents an important genomic platform that has great sensitivity and specificity, covers a wide dynamic range, and requires minute amounts of cells or tissue from which to isolate RNA. While qRT-PCR has significant diagnostic potential, it has to date been limited to viral diagnostics. Genomic Health Inc. (GHI) in collaboration with National Surgical Adjuvant Breast and Bowel Project (NSABP) researchers have recently developed and commercialized a predictive gene signature-based assay for ER-positive, LN-negative tamoxifen-treated breast cancer tumours, named Oncotype DX [28], measuring the expression of 21 genes on archival FFPE pathology blocks. GHI/NSABP researchers studied 447 patients from 3 independent clinical studies to test the relationship between expression of 250 candidate cancer-related genes (selected from published sources and public databases) and recurrence. They subsequently derived their gene list (16 genes plus 5 reference genes) and recurrence score (RS) algorithm which was prospectively tested on 668 patients enrolled in the NSABP trial B-14 and was found to provide accuracy and precision in predicting the likelihood of distant recurrence. Moreover, the RS performance exceeded standard measures such as age, tumour size, and tumour grade, both in prognostic power and in reproducibility [28]; this technology has been recently approved by the US Food and Drug Administration for clinical application.

Since then, this technique has undergone extensive testing regarding validation and prognostic capabilities: 1) the previously described RS has been shown to predict response to chemotherapy [57]. This work demonstrated that a higher RS is associated with a higher likelihood of pathologic complete response in patients treated with doxorubicin/paclitaxel neoadjuvant therapy in locally advanced breast cancer; 2) RS was shown to be not only prognostic for tamoxifen-treated patients, but also strongly predictive of response and benefit from tamoxifen in NSABP B-14 [28]; 3) moreover, RS predicted the magnitude of chemotherapy benefits in NSABP B-20: patients with tumours that had a low RS, derived minimal if any benefit from chemotherapy. Patients with tumours that had a high RS, derived a large absolute benefit from chemotherapy; 4) the initial B-14 prognostic data has been subsequently confirmed in a validation study of 220 evaluable cases and 570 matched controls in the Northern California Kaiser Permanente [58]. RS was strongly prognostic of breast cancer specific mortality in this population similar to the B-14 population. Finally, 5) the RS results from NSABP B-14 were correlated and compared with 10-year outcome data estimated using AO [59]. RS and AO predicted outcomes correlated relatively weakly (concordance = 48%) with RS appearing to correlate more strongly with outcome than AO. Hence, each algorithm/assay clearly contains independent prognostic information; it would, therefore, be reasonable to combine these information sets in future prognostic and predictive algorithms.

Clearly, the ability to validate a set of biomarkers on large FFPE sample sets is what allowed this assay to go forward and move into the clinic. Newer technology, such as the one discussed below, would allow for the analysis of large sets of potential biomarkers using RNA prepared from FFPE tumour tissue.

## The DASL assay

The DASL assay from Illumina Inc. has been specifically designed as a gene expression profiling system to generate reproducible data from degraded RNAs such as those derived from FFPE tumour samples. The assay is a cross between microarray and qRT-PCR technologies and endeavors to combine them into one platform that can be formatted to analyze expression of a panel of selected genes in a single clinical sample using a minimal amount of total RNA (≤ 200 ng total RNA per assay).

Potential advantages of DASL over other competing expression technologies include; 1) use of FFPE tissue samples as old as 24 years (unpublished data, Illumina); 2) high throughput, up to 96 clinical samples on one array plate; and 3) the use of a custom gene panel, at least 512 genes per array.

**Figure 2:**
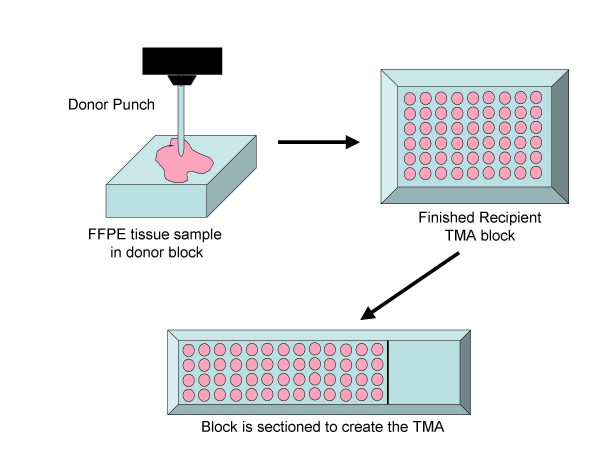
How TMAs are constructed. Cores (0.6–2.0 mm in diameter) are taken from FFPE tumour tissue donor blocks and arrayed into a recipient block. The recipient block is then sectioned (5 μm) onto a glass slide and processed by immunohistochemistry for biomarkers. The analysis can be manual or automated and the data is linked to clinical information.

Each array contains 50,000 beads with 1,534 different addresses and 3 unique addresses per gene. Each bead has hundreds of thousands of capture probes (23-mers) on fibers with one unique address. Therefore, analysis of ~500 genes per array panel can be conducted at ~30-fold redundancy (Fig. 2). Oligonucleotides are designed such that there are three non-overlapping probe pairs per gene. This results in a 1,506-plex measurement for each sample. It has been shown that using this number of probes per gene lends the assay the necessary sensitivity and reproducibility for quantitative detection of differential expression using RNA from FFPE tissues [8,60]. In the procedure, random priming is used for cDNA synthesis and, therefore, probes are designed such that they can target any unique region of the gene without limiting the selection of the optimal probe to the 3' ends of transcripts. In addition, due to the small size of the targeted gene sequence (50 nucleotides), along with the use of random primers in the cDNA synthesis, this allows for detection of RNAs that are otherwise too degraded for conventional microarray analysis.

The 5' oligonucleotides consist of two parts: the gene specific sequence and a universal PCR primer sequence. The 3'-oligos consist of three parts: the gene specific sequence, a unique address sequence which is complementary to one of 1,506 capture sequences on the array and a universal PCR primer sequence at the 3' end. A single address sequence is uniquely associated with a single target site. This address sequence allows the PCR-amplified products to hybridize to a universal microarray bearing the complementary address sequences.

In the report by Bibikova et al (2004), they showed that the DASL assay could be applied to breast and colon cancer FFPE tumor samples. Using a 231 gene cancer panel and cluster analysis, they were able to separate breast from colon tissue types and subsequently, divide each tissue sample set into cancer versus normal. The goal, therefore, is to use FFPE tumour tissue samples in conjunction with the DASL platform to potentially identify differential signature gene sets that can be used for diagnosis, prognosis and/or monitoring of disease. This technology is just beginning to be applied to cancer research and as its use becomes more widespread it has the potential to have an important impact on translational breast cancer research.

## Proteomic analysis of serum in breast cancer

**Figure 3:**
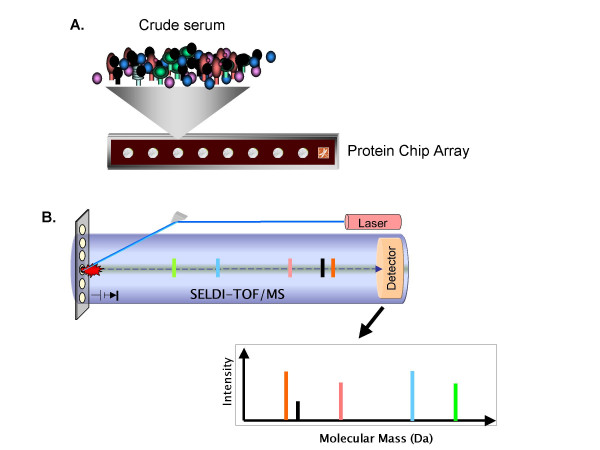
Analysis of serum proteins using ProteinChip arrays and SELDI-TOF/MS. **A. **Crude serum sample is placed (and processed) on a ProteinChip array which contains chemically (cationic, anionic, hydrophobic, hydrophilic, etc.) or biologically treated surfaces for specific interaction with proteins of interest. Proteins, thus, bind to chemical or biological "docking sites" on the ProteinChip surface. Non-binding proteins, salts, and other contaminants are washed away, eliminating sample "noise". **B. **Retained proteins are "eluted" from the ProteinChip array by Surface-Enhanced Laser Desorption/Ionization (SELDI). Ionized proteins are detected and their mass accurately determined by Time-of-Flight Mass Spectrometry (TOF/MS).

The ability to detect cancer early before it has metastasized throughout the body is one of the keys to ensuring that treatment has the highest likelihood of effectuating a complete cure. Of equal importance is the potential to monitor a patient's response to therapy or the potential of recurrence in real-time. What is required, therefore, is a reliable non-invasive diagnostic test. Serum has the advantage of being a readily accessible body fluid that is protein-rich and that is well-suited to proteomic analysis and hence, biomarker discovery or monitoring of a patient's condition with time. As an approach to serum biomarker discovery, proteomic pattern analysis has been developed as a means to identify novel markers when comparing samples from patients with disease with those from healthy subjects without any prior knowledge or bias of what the proteins are [61]. Interesting peaks can then be subsequently identified and confirmed as potential biomarkers. Differences in proteomic patterns in serum of diseased compared to normal can be due to; 1) overexpression; 2) abnormally shed proteins or protein fragments; 3) modified proteins; 4) proteolytically cleaved proteins; or 5) degradation due to the proteosome pathway. Only a small amount of serum, 1–20 μL, is required for analysis. Samples are added to protein-chip arrays, available in a number of different chromatographic surfaces, which are used to capture proteins based on charge or hydrophobicity. The retained proteins are then subjected to surface-enhanced laser-desorption ionization time-of-flight mass spectrometry (SELDI-TOF/MS), a proteomic platform amenable to high throughput analysis of serum samples (Fig. 3) (for a review of the technology see [62]).

An early study to find biomarkers in serum of breast cancer patients using SELDI-TOF/MS and Ciphergen ProteinChip^R ^arrays was undertaken by Li and colleagues [63]. They studied serum samples from 103 breast cancer patients at different clinical stages (stage 0 to III); 41 healthy women and 25 women with benign breast diseases. They identified three biomarkers that could discriminate breast cancer patients from the noncancer patients with high sensitivity (93%) and high specificity (91%). These three biomarkers were selected using stage 0-I cancer versus noncancer controls as the training set and later-stage cancer as the test set in order to try and identify early-stage breast cancer biomarkers. The biomarkers, however, were not sensitive to the stages of cancer patients. Therefore, these markers appear to reflect the malignant nature of the tumour rather than its progression. Similar results were obtained in a study of serum proteins from 49 breast cancer patients, 51 patients with benign breast diseases and 33 healthy women [64]. Using a combination of proteomics and bioinformatics, four candidate biomarkers of breast cancer were found and were used in the development of diagnostic models. The four putative markers could be used to distinguish between breast cancer patients and women who were healthy or had benign breast diseases.

SELDI-TOF/MS analysis of serum proteins can also be potentially applied to identify patients who would benefit from various breast cancer therapies as well as those who would experience adverse effects caused by chemotherapies used in breast cancer. In one such study, proteins with molecular weights of 7790 and 9285 Da were detected in 5 patients treated with docetaxel but were not detected in one patient who experienced severe, acute adverse effects [65]. Pusztai and colleagues [66] applied SELDI-TOF/MS to investigate proteomic changes in plasma from 69 Stage I-III breast carcinoma patients treated with paclitaxel or 5-fluorouracil, doxorubicin, and cyclophosphamide (FAC) chemotherapy and 15 healthy subjects. They detected a single chemotherapy-induced peak that did not, however, correlate with the tumour response, as well as five additional peaks that could distinguish breast carcinoma patients from normal healthy women.

As with many of these initial studies, further standardization and independent validation using larger numbers of specimens is required to ensure the performance of these selected biomarkers [67]. While a great deal more has to be done to validate potential biomarkers discovered in the serum from breast cancer patients, the potential is there to combine these biomarker analyses with other diagnostic procedures for the early detection and monitoring of response to therapy of breast cancer patients.

## A systems biology approach to data integration

Cancer in general, and breast cancer in particular, is a highly complex and heterogeneous disease which involves a succession of genetic changes that eventually results in the conversion of normal cells into cancerous ones. Hanahan and Weinberg have proposed that human tumours are governed by a common set of six acquired capabilities; 1) self-sufficiency in growth signals; 2) insensitivity to anti-growth signals; 3) evasion of apoptosis; 4) limitless replicative potential; 5) sustained angiogenesis; and 6) tissue invasion and metastasis [68]. A complete knowledge of these processes will require the integration and analysis of massive amounts of data, as is being collected from current genomic and proteomic platforms, as well as newer technologies [69].

Although systems biology is an emerging field, progress is being made and a number of computational approaches have been applied to the biological complexity of cancer. Christopher and colleagues [70] have developed a computer simulation of a human cancer cell. These whole cell mathematical models integrate vast amounts of data that include many interacting genes, proteins and protein modifications. They created a model of networks, both signal transduction and gene expression, that are involved in the control of cell proliferation and apoptosis and showed that it could be used to test the efficacy of drugs as well as explore various therapeutic targets. Computational methods are also being applied to expression data, both genomic and proteomic, in order to develop graphical models of gene-protein regulatory networks [71]. A number of additional computational approaches are being used in order to incorporate and connect experimental data so that biological systems can be simulated and used for hypothesis testing [72].

As systems biology matures, data that has been collected from various "omic" platforms will be available for input into novel computational systems biology models that will help unravel the complexity of cancer. Applying this systems biology approach to breast cancer has the potential to more rapidly lead to early diagnosis and the individualization of treatment.

## Conclusion

Microarray analysis of breast tumour tissue samples has heralded in a new age of molecular classification of tumours that has resulted in the identification of specific subtypes yielding new insights into prediction of disease outcome and response to therapy. However, this genomic platform is currently limited, for the most part, to analyzing frozen tumour tissue specimens, which are not readily available, thus, preventing large scale validation studies to be conducted using this technology. FFPE tumour tissue samples are, therefore, the most important and abundant source of material available from randomized clinical trials that will allow for well-controlled hypothesis testing to be conducted.

Thus, technologies designed for use with FFPE samples are critical in order to test and validate predictive gene signatures and biomarkers derived from microarray studies. The ability to tap into this resource is starting to have an impact as seen in the application of the qRT-PCR-based Oncotype DX assay to breast cancer patients. TMAs are also being used in the analysis and validation of biomarkers discovered through microarrays. Newer technology, such as the DASL assay, which has recently become available, holds the promise of further expanding the utilization of these precious tissue resources for both testing and validation of potential biomarkers. In addition, protein-chip technology in conjunction with SELDI-TOF/MS has opened up the entire field of protein pattern analysis of biological fluids in general and serum in particular. This proteomic platform has shown promise in both the discovery of biomarkers for early diagnosis and monitoring of disease progression.

The road to individualization of treatment for breast cancer patients is not an easy one with many twists and turns that will require an understanding of copious amounts of data generated from both genomic and proteomic platforms. The integration of all of this data using a systems biology approach will also be crucial in extracting the necessary information that will eventually lead to a detailed understanding of breast cancer.

We are leaving a past where the patient received the best therapy based solely on historical clinical efficacy data obtained from large populations of patients but without any specific prediction of individual response. We are entering an era where each and every patient will receive individualized therapy based upon the key signaling pathways driving her tumour; we believe that a combined platform approach incorporating both genomics and proteomics in a systematic way is critical for moving towards this goal.

## Abbreviations

AO, adjuvant online

BCL2, B-cell CLL/lymphoma 2

BRCA1/2, breast cancer 1/2, early onset

CA IX, carbonic anhydrase IX

CD117, v-kit Hardy-Zuckerman 4 feline sarcoma viral oncogene homolog

CDK, cyclin D kinase

CK5/6, cytokeratin 5/6;

c-Myc, v-myc myelocytomatosis viral oncogene homolog

Cox-2, cyclooxygenase-2

DASL, cDNA-mediated Annealing, Selection, extension and Ligation

DFS, disease-free survival

EGFR, epidermal growth factor receptor 1

ER, estrogen receptor

FFPE, formalin-fixed paraffin-embedded

FISH, fluorescence *in situ *hybridization

GHI, Genomic Health Incorporated

HDAC, histone deacetylase

HER2, epidermal growth factor receptor 2

Ki67, antigen identified by monoclonal antibody Ki-67

LN, lymph node

MINDACT, Microarray for Node Negative Disease may Avoid Chemotherapy

MUC, mucin

NSABP, National Surgical Adjuvant Breast and Bowel Project

NSAID, non-steroidal anti-inflammatory drug

OS, overall survival

p16, cyclin-dependent kinase inhibitor 2A

p21, cyclin-dependent kinase inhibitor 1A

p27, cyclin-dependent kinase inhibitor 1B

p53, tumour suppressor protein p53

p63, tumour protein p63

PGR, progesterone receptor

PTEN, phosphatase and tensin homolog

qRT-PCR, quantitative reverse transcriptase polymerase chain reaction

RS, recurrence score

SELDI-TOF/MS, surface-enhanced laser-desorption ionization time-of-flight mass spectrometry

TMA, tissue microarray

## Competing interests

The author(s) declare that they have no competing interests.

## Authors' contributions

MA and BLJ co-wrote the review and approved the final manuscript.
